# Peripheral Regulatory T Cells Display Dynamic Memory Subset Frequency and Inhibitory Marker Expression Across Pregnancy

**DOI:** 10.1111/aji.70238

**Published:** 2026-04-26

**Authors:** Nolawit Mulugeta, M. Quinn Peters, Cara Tobey, Jia Wong, Abigail L. P. Spray, Blair Armistead, Yonghou Jiang, Ronit Katz, Raj Shree, Lucia Vojtech, Whitney E. Harrington

**Affiliations:** ^1^ Center For Global Infectious Disease Research Seattle Children's Research Institute Seattle, WA USA; ^2^ Department of Global Health University of Washington Seattle, WA USA; ^3^ Department of Obstetrics & Gynecology University of Washington School of Medicine Seattle, WA USA; ^4^ Department of Pediatrics University of Washington School of Medicine Seattle, WA USA

## Abstract

**Problem:**

Pregnancy is characterized by significant changes in peripheral immunity. Total CD3+ T cells decrease across pregnancy while Forkhead Box P3+ T‐regulatory cells (FoxP3+Tregs) peak in mid‐pregnancy, the latter of which are key to healthy outcomes. However, less is known regarding distinct memory populations of FoxP3+ Tregs and their phenotypic changes across pregnancy.

**Method Of Study:**

Pregnant individuals were enrolled into a prospective cohort study and high parameter flow cytometry was used to characterize peripheral blood mononuclear cells collected at first and second trimester and delivery. Memory and phenotypic marker expression on FoxP3+ Tregs at each timepoint were analyzed. Non‐Treg CD4+ T cells and CD8+ Tcells were assessed as comparison populations. Suppressive capacity of FoxP3+ Tregs was compared between first trimester and delivery.

**Results:**

The frequency of central and effector memory populations within FoxP3+ Tregs, non‐Treg CD4+ T cells, and CD8+ T cells decreased across pregnancy (*p* ≤ 0.001 for all). Inducible T cell Costimulator (ICOS) expression decreased on FoxP3+ Tregs across pregnancy (*p* = 0.01), while T cell immunoglobulin and mucin domain‐containing protein 3 (Tim3) expression increased on FoxP3 Tregs, non‐Treg CD4+, and CD8+ T cells (*p* ≤ 0.005 for all). Suppressive capacity of Tregs did not vary by gestational timepoint.

**Conclusions:**

We observed an increase in naïve cells in FoxP3+ and non‐Treg CD4+ T cells across gestation and a corresponding reduction in ICOS expression. However, the observed increase in Tim3 expression on all T cell subsets suggests a dissociation between suppressive markers in pregnancy. Together, these data suggest that pregnancy has a unique impact on Treg memory distribution and phenotype.

## 1 Introduction

Pregnancy is a time of dynamic immune remodeling, a process important for healthy outcomes. Normal pregnancy is characterized by successful transitions between inflammatory‐like states in early and late pregnancy with a pro‐tolerant state in mid‐pregnancy [1]. Disruption in these states is associated with adverse outcomes like preeclampsia and preterm birth [2, 3]. Dynamic changes in immunity occur both in the local uterine and placental environment, as well as within the peripheral immune system of the pregnant individual. Shifts in natural killer (NK) cell, macrophage, and T cell populations within the uterus and placenta are relatively well studied, however less is known about changes in the peripheral immune compartment across gestation [4, 5].

Prior work demonstrates that regulatory CD4+ CD25^high^CD127^low^FoxP3 expressing T cells (Tregs) play a key role in maintaining tolerance to the fetus [5, 6]. Like all T cells, Tregs emigrate from the thymus in a naïve state, and expand and differentiate into effector or central memory subsets following antigen encounter. In non‐pregnant individuals, naïve CD45RA‐expressing Tregs make up the largest peripheral Treg population [7]. By comparison, central memory T cells reside in lymphatics and can circulate in the blood, while effector memory cells are typically located in tissue, including the decidua, and can also be found circulating in blood [8]. Memory Tregs have stronger and more rapid immunosuppressive functions compared to naïve Tregs, thus changes in Treg memory subsets may reflect varied functional capacity of the overall Treg pool [9, 10]. In the mouse model, memory Tregs specific for fetal antigens accumulate during pregnancy, persist after parturition, and expand more rapidly upon successive conceptions, providing an additional level of protection against complications related to disrupted fetal tolerance [11]. During pregnancy, a number of studies have shown that overall peripheral Treg numbers are dynamic throughout gestation, increasing until mid‐pregnancy and then decreasing close to labor [12, 13, 14, 15, 16]. Less is known about changes in Treg central memory versus effector memory or functional subsets throughout pregnancy with a few studies finding that the proportion of overall memory subsets decreases in the third trimester with a corresponding increase in naïve Treg frequencies [17, 18, 19]. Variations in these patterns have been linked to pregnancy complications. For example, in early pregnancy failure compared to uncomplicated pregnant controls, the peripheral Treg pool may have a lower frequency of naïve Tregs, whereas in preeclampsia, memory Tregs remain a higher proportion of the peripheral Treg pool [17, 20].

The expression of specific surface proteins on Tregs can indicate their activation status and functional potential, including capacity for suppression and communication with dendritic cells, effector T cells, and non‐immune cells [21, 22, 23, 24, 25]. However, a clear picture of functional marker expression on peripheral Treg subsets during pregnancy is still lacking, partly because prior studies have utilized variable populations and sampling timelines and a less specific definition of Tregs (CD4+ CD25+ cells) that has since been improved to include FoxP3 [6, 9]. In some studies, lower expression of immunosuppressive markers including cytotoxic lymphocyte associated protein 4 (CTLA‐4), HLA‐G, and programmed death protein 1 (PD‐1) on Tregs have been linked with pregnancy complications including recurrent pregnancy loss, miscarriage, gestational diabetes, and preeclampsia [18, 26, 27, 28, 29]. Thus, a greater understanding of how memory subset dynamics and functional markers change over gestation is critical to increasing our understanding of how Tregs support healthy pregnancies.

In this study, we used high parameter flow cytometry to investigate the frequency and functional profile of FoxP3+ Tregs in peripheral blood from individuals at three timepoints during pregnancy. Our results demonstrate dynamic changes in memory Treg pools over gestation and provide novel insight into the functional marker expression patterns of peripheral FoxP3+ Tregs.

## 2 Methods

### 2.1 Cohort

Individuals aged 18–45 with singleton pregnancies receiving obstetric care at the University of Washington affiliated obstetrics clinics were approached for enrollment into the Fetal Immune Development Study at the time of their first prenatal visit. Exclusion criteria were multifetal gestation, pregnancy‐ or placental‐associated complication, fetal aneuploidy, or primary or secondary immunodeficiency or modulation. Whole blood was collected at routine prenatal visits during the first (8‐12 weeks gestation) and second (24‐28 weeks gestation) trimesters and at the time of delivery. Peripheral blood mononuclear cells (PBMCs) were isolated ​​from whole blood as previously described and stored long term in liquid nitrogen [30]. Clinical data including maternal age, pregnancy history, parity, prior and current pregnancy related health conditions, and medications were abstracted from the medical chart following delivery. Individuals who developed placental‐associated conditions or preterm labor were excluded from analysis. All participants provided written informed consent, and the study was approved by the UW IRB (protocols STUDY00001636, STUDY00004878). Study data were collected and managed using REDCap electronic data capture tools hosted at University of Washington [31].

### 2.2 Flow Cytometry

On the day of experiment, PBMC were thawed and resuspended in thawing media (RPMI with L‐glutamine, 20% FBS) at 37°C. PBMCs were then pelleted at 400g for 5 minutes and resuspended in complete media (RPMI with L‐glutamine, 10% FBS, 100 U/ml penicillin and 100 µg/ml streptomycin), followed by counting. Cells were then stained with Fixable U/V Blue Live Dead Stain (Invitrogen) and Fc Receptor Blocking Solution (Biolegend) for 15 minutes, followed by staining with the extracellular antibody cocktail solution for 30 minutes at room temperature. Samples were then fixed and permeabilized for 40 minutes with the Foxp3/Transcription Factor Staining Buffer Set (ThermoFisher). Following permeabilization, the intranuclear staining cocktail was added for 30 minutes and samples were washed twice and resuspended in FACS (PBS with 2–5% FBS) buffer. Samples were then acquired on a FACSymphony A5 (BD Biosciences). The complete antibody panel was based on a previously validated and published 28 color T cell phenotyping panel (Table S1) [32]. All samples from a single individual were run on the same day and a technical replicate control sample was also included in each experiment. Voltages were calibrated daily using Rainbow Calibration Particles (Spherotech). Single stained compensation controls were made daily using the BD CompBeads Set Anti‐Mouse and Anti‐Rat beads (ThermoFisher) and ArC Amine Reactive Compensation Bead Kit (ThermoFisher).

### 2.3 Boolean Gating Analysis in Flowjo

**FIGURE 1:**
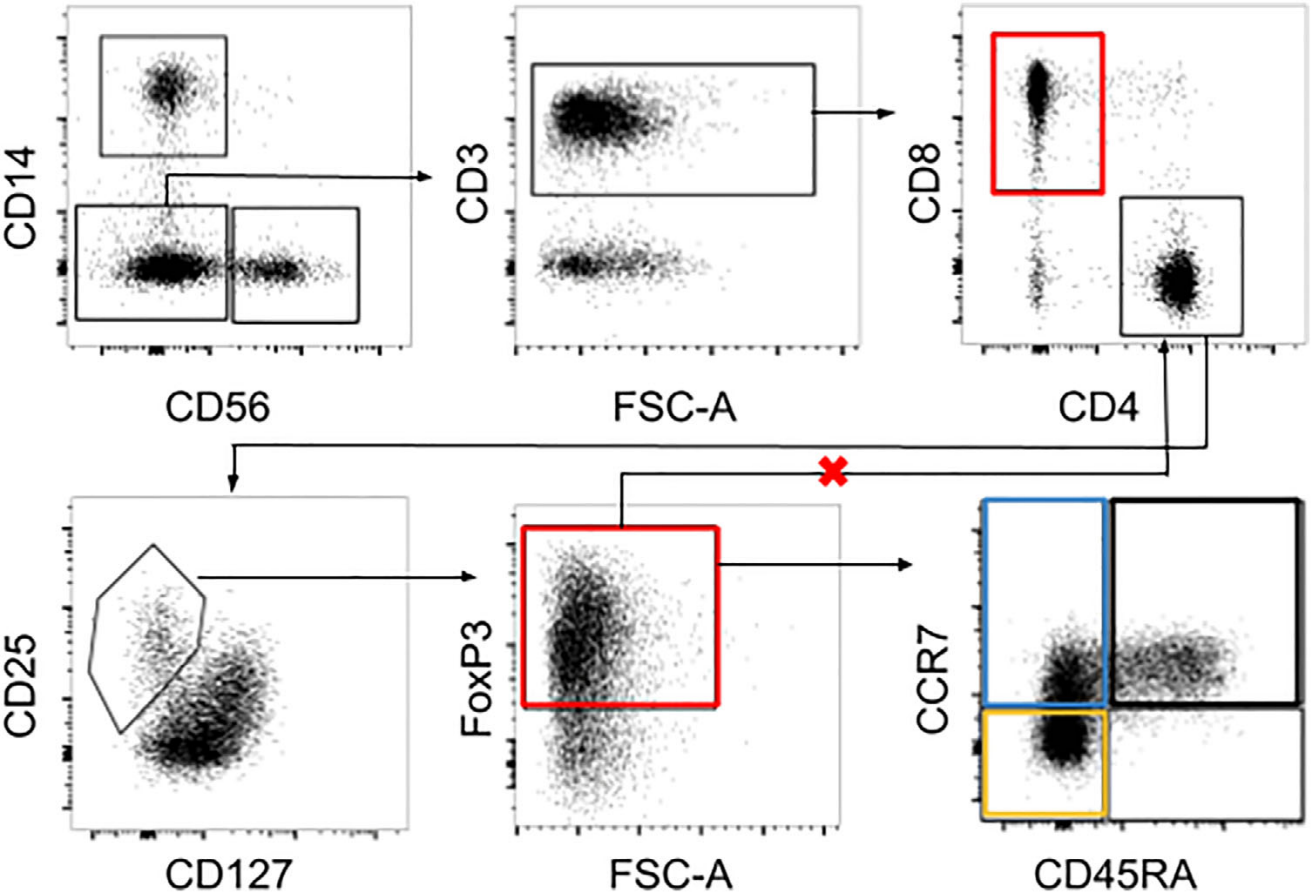
Peripheral blood mononuclear cells across gestation were analyzed with high dimensional flow cytometry. Gating tree from live single cells including CD14+ monocytes, CD56+ NK cells, and CD3+ T cells. Amongst CD3+ T cells, we identified CD8+ and CD4+ populations. CD4+ T cells were further sub‐gated as FoxP3+ Tregs and non‐Tregs (all CD4+ T cells that were not in the Treg gate). Tregs, non‐Treg CD4+ T cells, and CD8+ T cells (populations indicated in red) were further gated into memory populations: naive (CCR7+ and CD45RA+) (black box), central memory (CCR7+ and CD45RA‐) (blue box), and effector memory (CCR7‐ and CD45RA‐) (yellow box) subsets. CD8 T cells were additionally gated for TEMRA (CCR7‐ CD45RA‐).

Gating was adjusted using a technical replicate PBMC sample run in each experiment and performed in FlowJo v10.10. Using the gating tree shown (Figure 1), live single cells were gated into CD14+ monocytes, CD56+ NK cells, and CD14‐/CD56‐ CD3+ T cells, the latter of which were further separated into a CD4+ CD25^hi^CD127^lo^FoxP3+ Treg population (hereafter referred to as FoxP3+ Tregs), non‐Treg CD4+ T cells defined as all CD4+ cells not falling within the FoxP3+ Treg gate, and a CD8+ T cell population. Within individual samples, populations with less than 100 cells were excluded from further downstream analysis (e.g., memory phenotyping). Memory subpopulations for CD4+ T cell populations were gated as naive (CCR7+ CD45RA+), central memory (CCR7+ CD45RA‐), or effector memory (CCR7‐ CD45RA‐). For CD8+ cells, the additional population of T effector memory RA+ (TEMRA, CCR7‐ CD45RA+) was also considered.

### 2.4 High Dimensional Unsupervised Analysis in Catalyst

For high dimensional analysis, identified populations of FoxP3+ Tregs, non‐Treg CD4+ T cells, and CD8+ T cells were batch corrected using cyCombine using scaled normalization without performing asinh transformation [33]. Following batch correction, a mean fluorescence intensity (MFI) of each non‐lineage marker defined as mean of batch corrected and scaled arbitrary fluorescence units (AU) was calculated for each sample. These MFI values were then utilized in a principal component analysis (PCA) or analyzed for changes across time. In addition, all individuals’ FoxP3+ Tregs or non‐Treg CD4+ T cells or CD8+ T cells were combined into a single cell experiment object in R and clustered using the CATALYST cluster function which performs clustering with flowSOM and meta clustering with ConsensusClusterPlus. Most stable cluster number (k) for each dataset was selected based on cluster stability, identified as the point at which the increase in area under the cumulative distribution function approached zero per ConsensusClusterPlus protocol [34]. Uniform Manifold Approximation and Prediction (UMAP) was performed in CATALYST using default parameters and visualized with cluster labels. Heatmaps were generated with normalized marker expression and scaled between 0–1, and both marker and population dendrograms were generated with average linkage of Euclidean distance.

### 2.5 Treg‐Mediated Suppression of Proliferation

We identified 13 participants with a sufficient number of PBMC from both first trimester and delivery timepoints to conduct functional studies. CD4+CD127lowCD25+ putative Tregs and CD4+CD25‐ responder T cells (Tresp) were isolated separately using EasySep Human CD4+CD127lowCD25+ Regulatory T Cell Isolation Kit (Stem Cell). Isolated Tresp were stained with CellTrace Violet Cell Proliferation Kit for flow cytometry (Invitrogen) according to the manufacturer instructions. For both negative and positive control conditions 10,000‐50,000 Tresp cells alone were added and for the positive control, Dynabeads Human T‐Activator CD3/CD28 for T Cell Expansion and Activation (ThermoFisher) were added in a 1:10 bead to cell ratio. For the experimental condition, the Tregs and CTV‐stained Tresp were mixed in a ratio of one Treg to two Tresp for a total of 30,000‐150,000 cells per participant depending on yield, and Dynabeads were added at a 1:10 bead to cell ratio. The media was supplemented with 150 U/ml of IL‐2 and incubated for three days at 37°C. Following incubation, cells were stained with Zombie UV Fixable Viability Kit (Biolegend) and Fc Receptor Blocking Solution (Biolegend) for 15 minutes. Data were analyzed with the FlowJo V10.10 proliferation tool. One sample did not yield enough cells to fit the proliferation model leaving 12 paired samples. For the proliferation tool, the undivided peak was fixed using the unstimulated CTV‐stained Tresp negative control for each sample and either 6 or 7 peaks were selected in the experimental condition, depending on observed peaks in proliferated samples. The proliferation index for each sample was then used to calculate a percent change in proliferation from Tresp alone to Tresp with Tregs for each sample.

### 2.6 Statistical Analysis

Clinical characteristics (Table 1) were summarized using the median and range for continuous variables. Effect of gestational age in weeks on population frequencies and cell surface marker expression was modeled using linear mixed effect models with the lme4 package in R. Gestational age and parity were included as fixed effects in all models. Participant ID and gestational age were included as non‐correlated random effects to account for individual variation in baseline levels and longitudinal trends across time. P‐values for the gestational age effect were obtained using the *mixed* function from the *afex* package in R. For proliferation studies, paired t tests were used to compare samples from first trimester to delivery. For all analyses p‐values < 0.05 were considered statistically significant. All analyses were performed with R version 4.4.0 (https://www.R‐project.org/).

**TABLE 1:** Demographic characteristics of participants.

Characteristic	Median (Range)
Age (years)	34 (23,44)
Gravidity	2 (1,6)
Parity	0.5 (0,2)
Gestational age at delivery (weeks)	40 (36,41)

## 3 Results

### 3.1 Demographic Characteristics

For the present sub‐study, we included 20 participants. However, one individual developed preeclampsia and was excluded from analysis, and one maternal‐fetal pair developed tachycardia at delivery concerning for infection, leaving n = 18 individuals with complete data. The median age of individuals was 34 (range 23, 44) (Table 1). Median gravidity was 2 (range 1,6) and median parity was 0.5 (range 0, 2). Sixteen individuals had no preexisting health conditions while one participant each reported Hashimoto's Disease and chronic hypertension managed without medication. In their current pregnancy, two individuals developed gestational diabetes mellitus, one diet controlled and one well controlled on metformin and low‐dose insulin (Table S2). Delivery occurred at a median gestational age of 40 weeks (range 36, 41).

### 3.2 CD3+ T Cells Decrease Across Pregnancy While CD14+ Monocytes Increase

**FIGURE 2:**
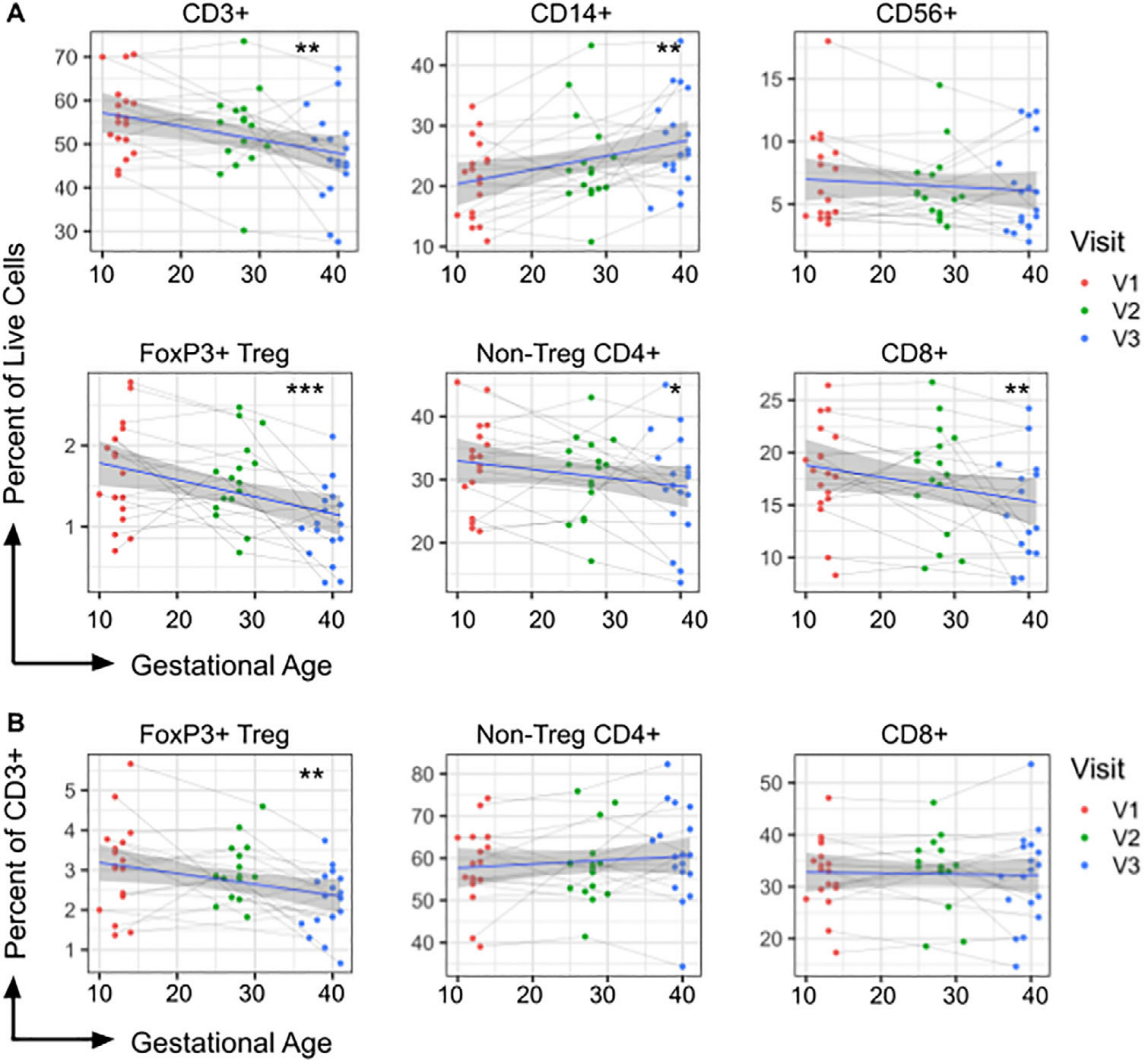
CD3+ T cells decrease as a percentage of live cells over gestation. Peripheral blood mononuclear cells were profiled using flow cytometry. (**A**) Frequency of CD3+ T cells, CD14+ monocytes, CD56+ NK cells, FoxP3+ Tregs, non‐Treg CD4+ T cells, and CD8+ T cells of live cells across gestation, (**B**) Frequency of FoxP3+ Tregs, non‐Treg CD4+ T cells, CD8+ T cells of CD3+ T cells across gestation is shown. Each data point represents an independent sample, and a simple linear regression model (*y*∼*x*) with a 95% CI is shown for visualization, with color indicating the visit number. Changes over time were modeled using linear mixed‐effects models, with *p*‐values reported for the effect of gestational age on the outcome indicated on *y*‐axis. *p ≤ 0.05, **p ≤ 0.01, ***p ≤ 0.001.

Within live cells, the frequency of CD3+ T cells, FoxP3+ Tregs, non‐Treg CD4+ T cells, and CD8+ T cells all significantly decreased across pregnancy (Figure 2A). The frequency of total CD3 T cells decreased by ‐0.3% per gestational week (GW) (p = 0.003), FoxP3+ Tregs by ‐0.022% per GW (p <0.001), non‐Treg CD4+ T cells by ‐0.1% per GW (p = 0.02), and CD8+ T cells by ‐0.12% per GW (p = 0.009) (Figure 2A). In contrast, the frequency of CD14+ monocytes in live single cells increased by 0.24% per GW (p = 0.002) (Figure 2A). The frequency of CD56+ NK cells amongst live cells did not significantly change (Figure 2A).

We also analyzed the changes in T cell subsets within parent CD3+ T cells and observed that FoxP3+ Tregs declined across pregnancy (‐0.03% per GW, p = 0.002) (Figure 2B). The frequency of non‐Treg CD4+ and CD8+ T cells amongst CD3+ T cells, as well as the CD4 to CD8 ratio, did not change significantly over the course of pregnancy (Figure 2B).

### 3.3 Naive Tregs Increase as a Proportion of Total FoxP3+ Tregs Across Pregnancy

**FIGURE 3:**
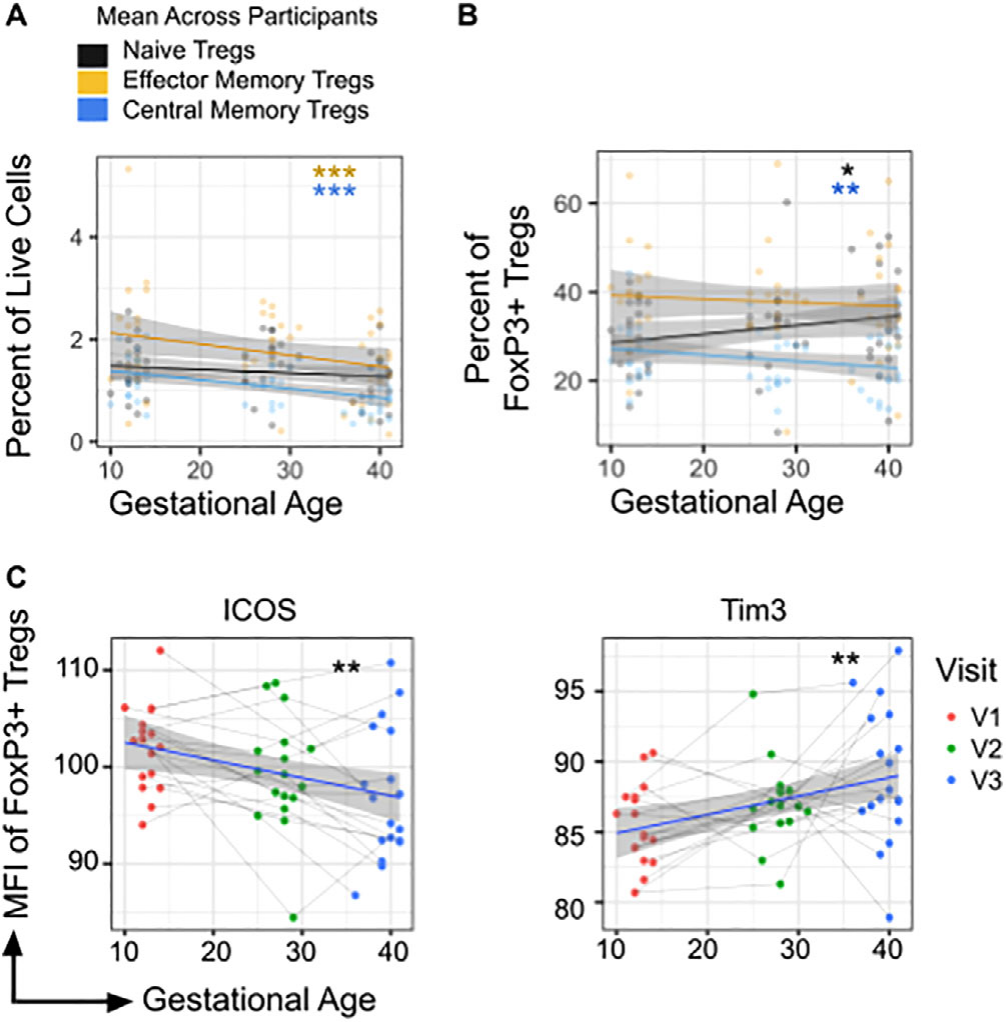
Peripheral FoxP3+ Treg compartment is dynamic over gestation. (A) Frequency of effector memory (CD45RA‐ CCR7‐), central memory (CD45RA‐ CCR7+) and naive Tregs (CD45RA+ CCR7+) of total live cells across gestation. (B) Treg memory subsets of total FoxP3+ Tregs across gestation. (C) MFI of ICOS and Tim3 on all FoxP3+ Tregs. Each data point represents an independent sample and a simple linear regression model (*y*∼*x*) with a 95% CI is shown for visualization, with color indicating visit. Changes across time were modeled using linear mixed‐effects models, and *p*‐values reflect the effect of gestational age on outcome shown on the y‐axis. *p ≤ 0.05, **p ≤ 0.01, ***p ≤ 0.001.

Because FoxP3+ Tregs decrease in live single cells across pregnancy, we assessed if this decrease was explained by a loss of specific FoxP3+ memory subsets. The frequency of both memory compartments in live single cells decreased across pregnancy, by ‐0.01% per GW for effector memory Tregs and by ‐0.007% per GW for central memory Tregs (p <.001 for both comparisons) (Figure 3A). In contrast, the change in frequency of naive Tregs in live cells did not reach statistical significance. This indicates that the decrease seen in frequency of FoxP3+ Tregs in live single cells was driven by a loss of memory Tregs.

To understand how loss of memory FoxP3+ Tregs in live cells affected the total FoxP3+ Treg pool, we assessed the dynamics of naive, central memory, and effector memory subsets within the parent FoxP3+ Treg population. As a proportion of total Tregs, naive Tregs increased 0.2% per GW (p = 0.039) while central memory Tregs decreased by ‐0.15% per GW (p = 0.002) (Figure 3B; Figure S1). Effector memory Tregs also decreased across pregnancy, although the change was not statistically significant. Together, these observations suggest that over gestation, the frequency of Tregs decreases and that naïve Tregs make up a greater proportion of the total Treg pool.

### 3.4 Expression of Cell Surface Markers Tim3 and ICOS on FoxP3+ Tregs is Dynamic Across Pregnancy

To understand phenotypic changes in FoxP3+ Tregs over pregnancy, we assessed the expression of key cell surface markers implicated in the inhibitory activity of Tregs. This included T cell immune checkpoint proteins PD1, CTLA4, TIGIT, and ICOS, tissue tolerance markers IL1R1 and Tim3, and the regulatory enzyme CD39 [22, 23, 24, 25]. Of these markers, ICOS expression on total FoxP3+ T cells decreased across pregnancy (p = 0.002), while Tim3 expression increased across pregnancy (p = 0.005) (Figure 3C). PD1, CTLA4, TIGIT, IL1R1, and CD39 expression did not change significantly over the course of pregnancy, although small decreases in expression were observed for some markers (Figure S2).

We next looked at the expression of these markers within Treg memory subsets. The decrease in ICOS expression was evident in all three subsets, though significant only in effector memory and naive FoxP3+ Tregs (p = 0.013 and p = 0.004 respectively, Figure S2). The increase in Tim3 expression, however, was statistically significant and of similar magnitude in all three memory compartments (Figure S2). Of the additional markers, CD39 decreased on effector memory Tregs only (p = 0.013) (Figure S2).

We finally assessed whether parity influenced the kinetics of any of these changes but did not identify any significant associations (Figure S3).

### 3.5 Unsupervised Analysis of Tregs Confirms Key Memory Subsets and Kinetic Changes Across Pregnancy

**FIGURE 4:**
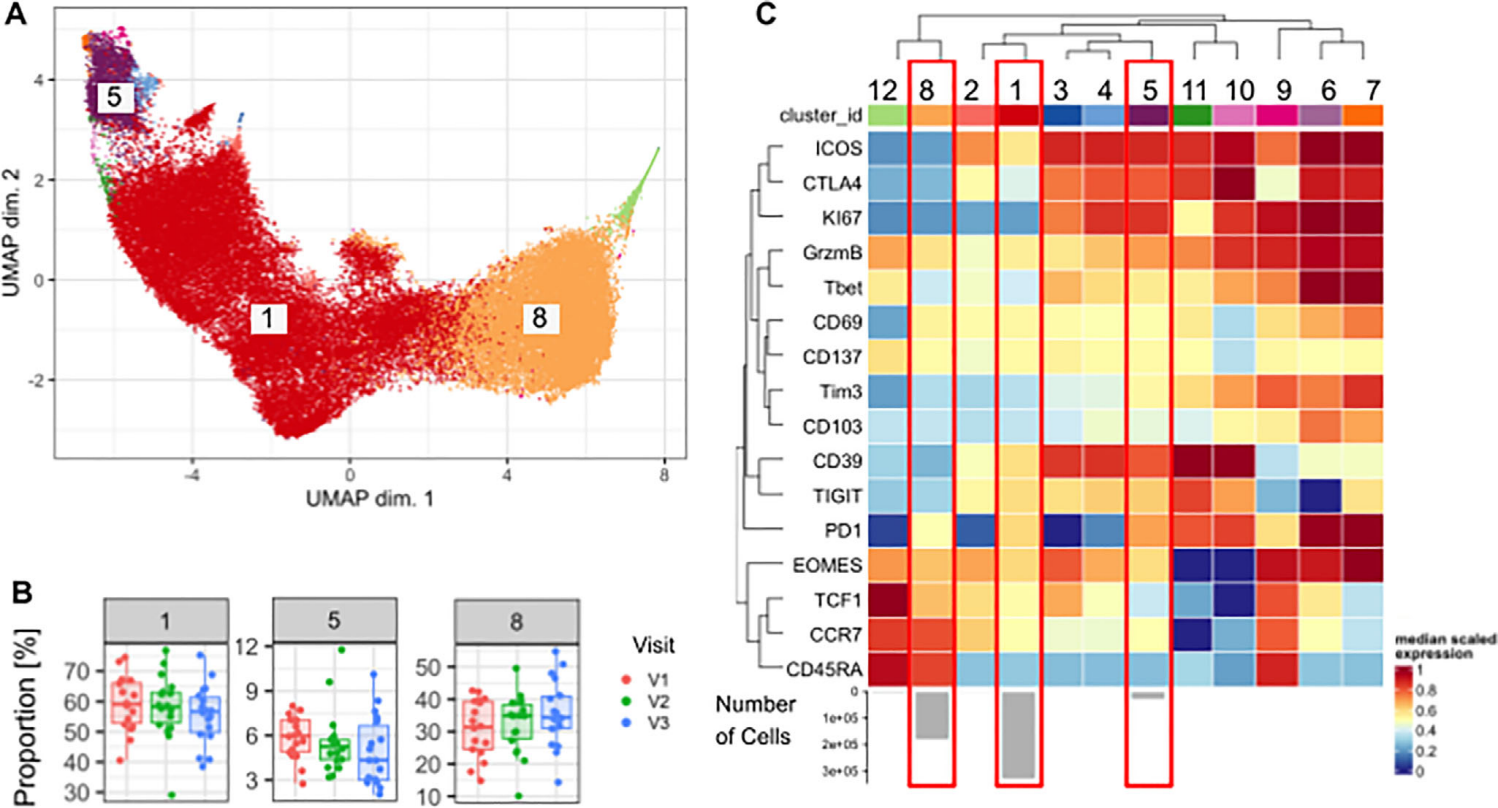
Unsupervised analysis confirms decrease of memory FoxP3+ Tregs across gestation. (A) UMAP representation of all FoxP3+ Tregs across all individuals with three key populations identified. Each data point represents a single cell. (B) flowSOM cluster frequency across visits. Each data point represents an independent sample. (C) Expression heatmap of phenotypic and functional markers.

Building on these findings, we sought to understand variation in Treg phenotype over time using an unsupervised approach. Using flowSOM unsupervised clustering and metaclustering with ConsensusClusterPlus, we identified 12 clusters (Figure 4). Of the top three clusters by frequency, only one, cluster 8, increased over pregnancy and expressed high levels of CCR7 and CD45RA, indicating that it was enriched for naive Tregs. In addition, this cluster also had expression of TCF1, a marker of stem‐like phenotype (Figure 4C).

The other two high frequency clusters decreased over the course of pregnancy. Of these, the most populous cluster in the data, cluster 1, expressed low CD45RA and intermediate CCR7 indicating a mix of central and effector memory Tregs. Compared to cluster 8, cluster 1 also expressed higher levels of ICOS, CTLA4, CD39, and TIGIT, consistent with prior antigen experience induced suppressive capacity. Lastly, cluster 5 which displayed the highest expression of ICOS, CTLA4, PD1, CD39 and KI67 suggestive of a suppressive, cycling memory subset, also decreased over pregnancy. This cluster was also farthest from the naïve‐like cluster on the UMAP plot indicating strong phenotypic difference from the naïve cluster.

### 3.6 Non‐Treg CD4+ T cells Show Similar Changes in Memory Phenotype Frequency

**FIGURE 5:**
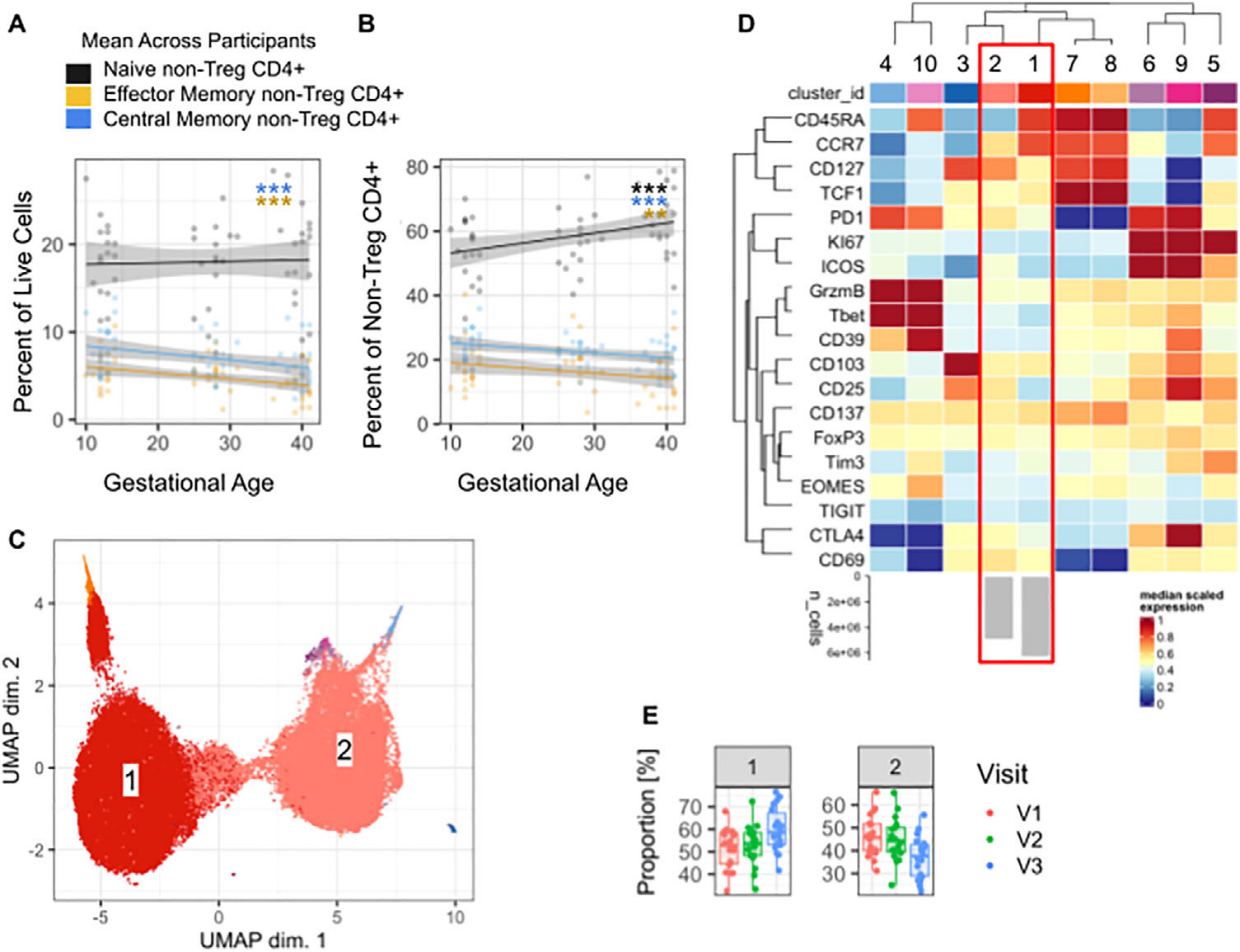
Peripheral memory non‐Treg CD4 T cells decrease across gestation. (A) Frequency of effector memory (CD45RA‐ CCR7‐), central memory (CD45RA‐ CCR7+) and naive populations (CD45RA+ CCR7+) of total live cells across gestation. (B) non‐Treg CD4+ T cell memory subsets of total non‐Treg CD4+ T cells across gestation. (C) UMAP representation of single cell non‐Treg CD4 T cells. (D) Phenotypic and functional marker expression heatmap. (E) Cluster frequency across visits. Each data point in (A, B, E) represents an independent sample and in (C) represents a single cell. For (A, B) a simple linear regression model (y∼x) with a 95% CI is shown for visualization, with color indicating visit. Changes across time were modeled using linear mixed‐effects models, and *p*‐values reflect the effect of gestational age on outcome shown on the y‐axis. *p ≤ 0.05, **p ≤ 0.01, ***p ≤ 0.001.

We next assessed whether the dynamic changes in memory Treg subset frequency across pregnancy were mirrored in the non‐Treg CD4+ population. Considered as a frequency of live cells, both effector memory and central memory non‐Treg CD4 T cells significantly decreased across pregnancy (‐0.06% per GW, p = <0.001; ‐0.08% per GW, p = 0.001), whereas the frequency of naive non‐Treg CD4 T cells did not change (Figure 5A). Out of total non‐Treg CD4+ T cells, both central and effector memory compartments decreased significantly (‐0.17% per GW, p = 0.01; ‐0.16% per GW, p <0.001), while naive cells increased (0.3% per GW, p <0.001) (Figure 5B).

Similar to Tregs, the expression of Tim3 increased significantly across gestation on non‐Treg CD4 T cells (p<0.001), while the expression of ICOS decreased (p = 0.002) (Figure S4). PD1 expression additionally decreased significantly across pregnancy (p = 0.025, Figure S4). Unsupervised analysis identified two broad clusters which together comprised over 95% of the non‐Treg CD4+ T cell pool (Figure 5C‐E). Cluster 1 increased across pregnancy and was characterized by high expression of CCR7 and CD45RA, suggesting enrichment for naive non‐Treg CD4 T cells. In contrast, cluster 2 showed lower expression of CD45RA and intermediate CCR7 expression suggesting a mixed memory phenotype. This cluster decreased across pregnancy and had higher expression of inhibitory markers ICOS, PD1, CTLA4; the activation marker CD25; and CD127 (Figure 5D).

### 3.7 CD8 T Cells Show More Muted Changes Across Pregnancy

**FIGURE 6:**
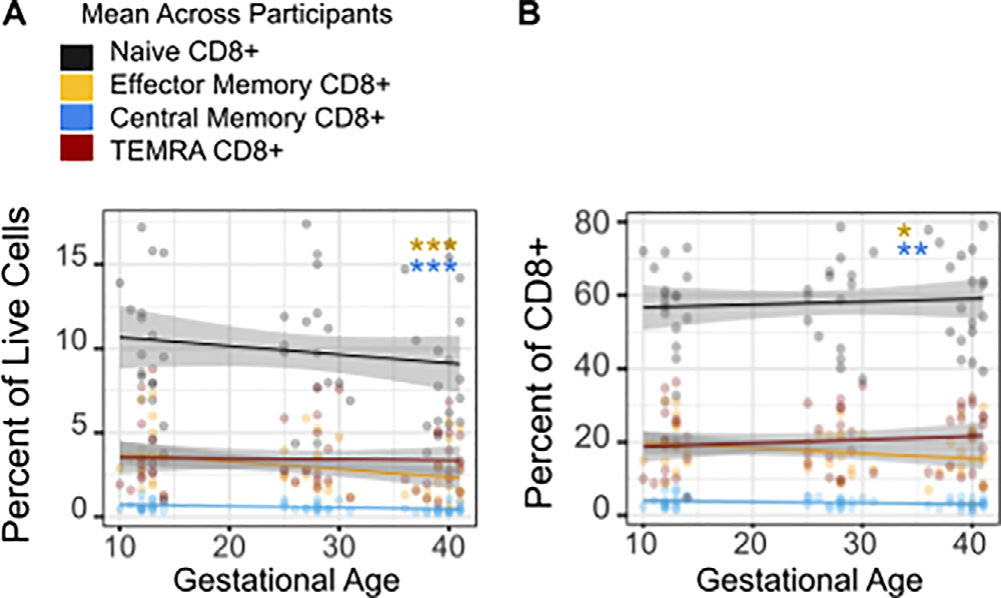
Peripheral memory CD8 T cells decrease across gestation. (A) Frequency of effector memory (CD45RA‐ CCR7‐), central memory (CD45RA‐ CCR7+), TEMRA (CD45RA+ CCR7‐), and naive populations (CD45RA+ CCR7+) of total live cells. (B) CD8+ T cell memory subsets of total CD8+ T cells. A simple linear regression model (y∼x) with a 95% CI is shown for visualization, with color indicating visit. Changes across time were modeled using linear mixed‐effect models, and with p‐values reflect the effect of gestational age on outcome shown on y‐axis. *p ≤ 0.05, **p ≤ 0.01, ***p ≤ 0.001.

As a frequency of live cells, both central and effector CD8+ T cells decreased across gestation (‐0.05% per GW, p<0.001; ‐0.01% per GW, p <0.001) (Figure 6A). Similarly, within total CD8+ T cells both central and effector memory CD8 T cells significantly decreased across pregnancy (‐0.03% per GW, p = 0.005; ‐0.17% per GW, p = 0.033) (Figure 6B). In contrast, there was no significant change in the frequency of naïve or TEMRA populations. Of the previously identified markers of interest, the effect of gestational age was significant only for an increase in expression of Tim3 (p = 0.002, Figure S4).

### 3.8 Treg Suppressive Function Does not Vary Across Gestation

**FIGURE 7:**
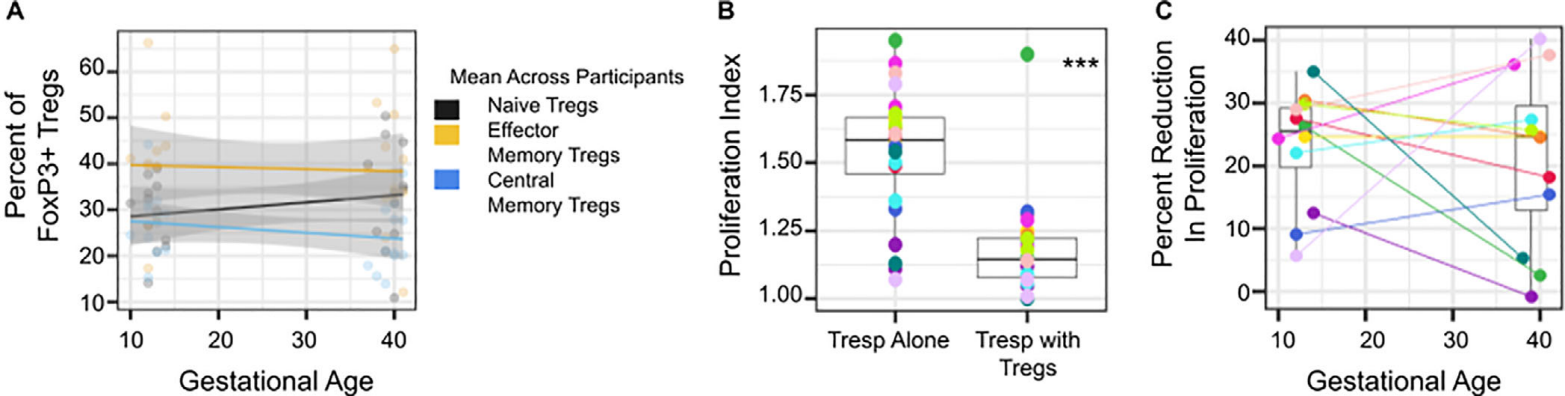
Treg suppressive function does not vary across gestation. (A) Frequency of effector memory (CD45RA‐ CCR7‐), central memory (CD45RA‐ CCR7+) and naive Tregs (CD45RA+ CCR7+) of total FoxP3+ Tregs across gestation for selected participants (*n* = 12). (B) Proliferation index of isolated Tresp cells with or without added Tregs. (C) Percent reduction in proliferation of Tresp with Tregs compared to Tresp alone for each participant and timepoint is shown. Colors indicate unique individuals. Paired t tests were used to compare groups, and p<0.05 was considered statistically significant. *p ≤ 0.05, **p ≤ 0.01, ***p ≤ 0.001.

To determine if the changes in memory populations and suppressive marker expression across gestation correlated with suppressive function, we tested the ability of Tregs to suppress proliferation of matched conventional CD4+ T cells (Tresp) in a subset of participants (Figure 7A). As expected, Tresp proliferated in the absence of Tregs (Mean Proliferation Index (MPI) of Tresp alone = 1.50, SD = 0.26), and this proliferation was significantly reduced in the presence of Tregs (MPI = 1.17 of Tresp with Tregs, SD = 0.17, p <0.001) (Figure 7B). We did not find a significant difference in the reduction of proliferation comparing first trimester to delivery samples (Mean % Reduction Proliferation Index at V1 = 23.0% versus V3 = 21.4 % p = 0.75) (Figure 7C). However, we did observe an association between the frequency of naive Tregs in each sample by initial flow cytometry phenotyping and a stronger reduction in proliferation in vitro (delta = 30% per 1% change in naive Treg frequency), which did not reach statistical significance (p = 0.23, Figure S5).

## 4 Discussion

In this study, we sought to comprehensively characterize shifts in T cell populations across pregnancy, with a particular focus on FoxP3+ Tregs. It has long been recognized that FoxP3+ Tregs play a key role in supporting healthy pregnancy [5, 6, 35]. More recent work has revealed substantial functional heterogeneity in Tregs, demonstrating that antigen encounters give rise to Treg memory populations and increased expression of functional markers such as CTLA4, Tim‐3, TIGIT, and ICOS [9, 10]. A memory phenotype and elevated expression of these markers alters Treg functional capacity through interactions with antigen‐presenting cells and increased capacity for IL‐10 secretion [36, 37]. The role of these markers on Tregs has been investigated at the maternal‐fetal interface, where PD1^hi^ and TIGIT‐expressing Tregs have been described [38]. However, changes in their expression patterns across gestation in the peripheral compartment remains poorly investigated. Here, we analyzed samples from the same individuals at three timepoints across gestation, enabling robust normalization for baseline inter‐individual variation and precise assessment of population shifts across gestation.

Consistent with prior studies, we observed a decrease in CD3+ T cells as a proportion of total live cells [39]. This reduction was evident across CD3+ subsets, including Tregs, non‐Treg CD4+ T cells, and CD8+ T cells and was driven largely by a contraction of memory cells. This points to a global mechanism affecting generation or maintenance of memory T cells in the periphery [40]. Memory T cells are known to traffic to local tissue sites more readily than their naïve counterparts [10]. The decidual environment is enriched for activated Tregs near term, suggesting that a gradual loss of memory Tregs from the periphery may reflect their migration to the maternal‐fetal interface [41]. Moreover, in preeclampsia and recurrent pregnancy loss, peripheral T cell populations more closely resemble those of non‐pregnant individuals, with higher frequencies of memory and activated T cell populations compared to those in healthy pregnancies [42, 43]. This pattern may reflect abnormal trafficking to the decidua. Future studies will be essential to elucidating the mechanisms governing T cell trafficking from the periphery to the maternal fetal interface in the context of both healthy and pathological pregnancy.

Given the increase in circulating fetal antigen as pregnancy progresses, one might expect an increase in memory Tregs in the periphery. Our findings, as well as prior work [44], reveal a more nuanced pattern across pregnancy with memory Tregs increasing from conception through the end of the first trimester, followed by a steady decline, accompanied by a concurrent rise in the proportion of peripheral naïve Tregs [18]. This dynamic may be altered in preeclampsia where the peripheral Treg pool contains fewer naïve Tregs and memory Tregs remain a higher proportion of the total [20]. Similarly, with assisted reproduction, an increase of naïve Tregs and decrease in memory Tregs in the periphery is associated with a higher chance of successful pregnancy [7]. Collectively, these findings suggest that maintenance of a dynamic balance between naïve and memory Treg populations is key for a healthy pregnancy outcome.

We were particularly interested in the expression patterns of phenotypic markers associated with Treg functional capacity across gestation. We assessed 14 functional markers, the majority of which did not change significantly across pregnancy, indicating peripheral T cells may be largely functionally stable over healthy gestation. One exception was an increase in Tim3 expression on all T cells, including Treg subsets and both non‐Treg CD4+ T cells and CD8+ T cells. Galectin‐9, the ligand for Tim3, is expressed by trophoblast cells and increases in circulation during pregnancy, possibly driving Tim3 upregulation [45]. This increase may be functionally important as Tim3+ Tregs are shown to be highly suppressive and tissue specific [36]. Furthermore, tolerizing CD8+ T cells and NK cells in the decidua express Tim3 [46, 47, 48], and reduced Tim3 expression in CD4+ T cells has been associated with recurrent pregnancy loss, implicating Tim3 upregulation in maintenance of healthy pregnancy [49].

In contrast to Tim3, ICOS expression decreased on FoxP3+ Tregs and non‐Treg CD4 T cells across gestation. This reduction may be partially explained by the relative contraction of memory Tregs in the periphery over pregnancy, as ICOS expression is upregulated on antigen‐experienced memory T cells [50]. However, we also observed decreased expression levels of ICOS within memory Treg populations across pregnancy and a particular loss on all CD4+ T cells, indicating more complex regulation. ICOS+ Tregs secrete more IL‐10 and reduce proinflammatory cytokine secretion in co‐cultured cells in vitro. [51] Consequently, our findings suggest that regulation of cytokine production by Tregs may shift toward allowing more inflammatory responses as pregnancy progresses.

Using conventional proliferation assays, we did not observe a consistent difference in suppressive capacity of Tregs between paired early and late gestation samples. However, these assays do not capture suppressive mechanisms mediated indirectly, such as interactions with antigen‐presenting cells or modulation of conventional T cell polarization [21]. Indeed, the most dynamic markers in this study, Tim3 and ICOS, are known to facilitate communication with antigen presenting cells and other non T cells [25, 52]. We also did not test any antigen‐specific Treg functions, which may play a role in pregnancy but are difficult to investigate. Interestingly, we observed a trend towards an association between suppression of proliferation and the frequency of naive Tregs in the total Treg pool, consistent with prior work showing that antigen‐experienced Tregs have less suppressive capacity [53]. This finding suggests increased naive Tregs later in gestation are poised to be more immunosuppressive in response to increased fetal allogeneic antigen load or other external immune stimuli.

In addition to contraction of memory CD4 T cells, we also observed a reduction in CD8 T cells. This finding is consistent with prior studies demonstrating that pregnancy is associated with a reduction in memory CD8 T cells and an increase in CD8+ T cells expressing killer cell immunoglobulin‐like receptors, which may contribute to suppression of allogeneic responses to fetal antigens [54]. Trafficking of adaptive immune cells from the periphery, particularly memory CD4 and CD8+ T cells, to the decidua could encourage tolerance through a local signal of ‘activation and dysfunction’ as has been reported for decidual CD8+ T cells [46]. In addition, we observed that the overall reduction in CD3+ T cells across pregnancy was accompanied by an increased proportion of CD14+ monocytes, consistent with other studies [39, 55, 56]. Monocyte subsets in pregnancy are shifted to M2‐like profile and exhibit more inflammatory phenotypes, which may reflect a response to fetal antigen in circulation [57, 58, 59] Together, these findings suggest that pregnancy is characterized by a contraction of memory adaptive populations alongside an increase in innate immune cells such as monocytes. Although work is ongoing to understand the underlying mechanisms, activation of innate immunity has been theorized to compensate for the loss of leukocytes in the peripheral blood whilst supporting healthy gestation [58, 60].

A major strength of our study was the longitudinal design which enabled adjustment for dynamic changes in cell population and marker expression in the same individual over time, an advantage compared to a cross‐sectional design. However, this study has several limitations. First, our sample size was somewhat small; despite this, we observed consistent changes in T cell population dynamics and phenotypes across pregnancy. Second, participants were enrolled at their first prenatal visit, precluding characterization of T cell populations in very early pregnancy. Third, because our flow cytometry panel was designed to characterize CD3+ T cell populations, we did not characterize phenotypic changes across other immune cell types, such as natural killer cells, which also play critical roles in pregnancy tolerance [4, 61]. Lastly, this study was focused on healthy pregnancy; future work will be important to determine whether the patterns we observed vary in pregnancy complications.

In summary, our data reveals that distinct aspects of peripheral immunity are dynamically regulated over normal human pregnancy. Peripheral T cell populations, including Tregs, are depleted of memory populations as pregnancy progresses. Concurrently, we find a selective increase in Tim3 and decrease in ICOS on both Treg and non‐Treg CD4 T cells, pointing to unique impact of pregnancy on peripheral T cell tolerance. Through in‐depth characterization of peripheral T cells across pregnancy, this study contributes to a more comprehensive understanding of the impact of pregnancy on peripheral immunity.

## Ethics Statement

The authors confirm that the ethical policies of the journal, as noted on the journal's author guidelines page, have been adhered to and the appropriate ethical review committee approval has been received. The study conformed to the US Federal Policy for the Protection of Human Subjects.

## Conflicts of Interest

The authors declare no conflict of interest.

## Supporting information




**Supplementary File 1**: aji70238‐sup‐0001‐SupMat.docx

**Supplementary File 2**: aji70238‐sup‐0002‐TableS3.xlsx

## Data Availability

The data that support the findings of this study are available in Supplementary File 2 (Table S3).
