# Supplementary material for: Peripheral Regulatory T Cells Display Dynamic Memory Subset Frequency and Inhibitory Marker Expression Across Pregnancy
Source: Am J Reprod Immunol. 2026 Apr 26;95:e70238. doi: 10.1111/aji.70238 (PMC13110828; doi:10.1111/aji.70238)
Supplement: Supplementary file 1 — aji70238‐sup‐0001‐SupMat.docx [file AJI-95-e70238-s002.docx]

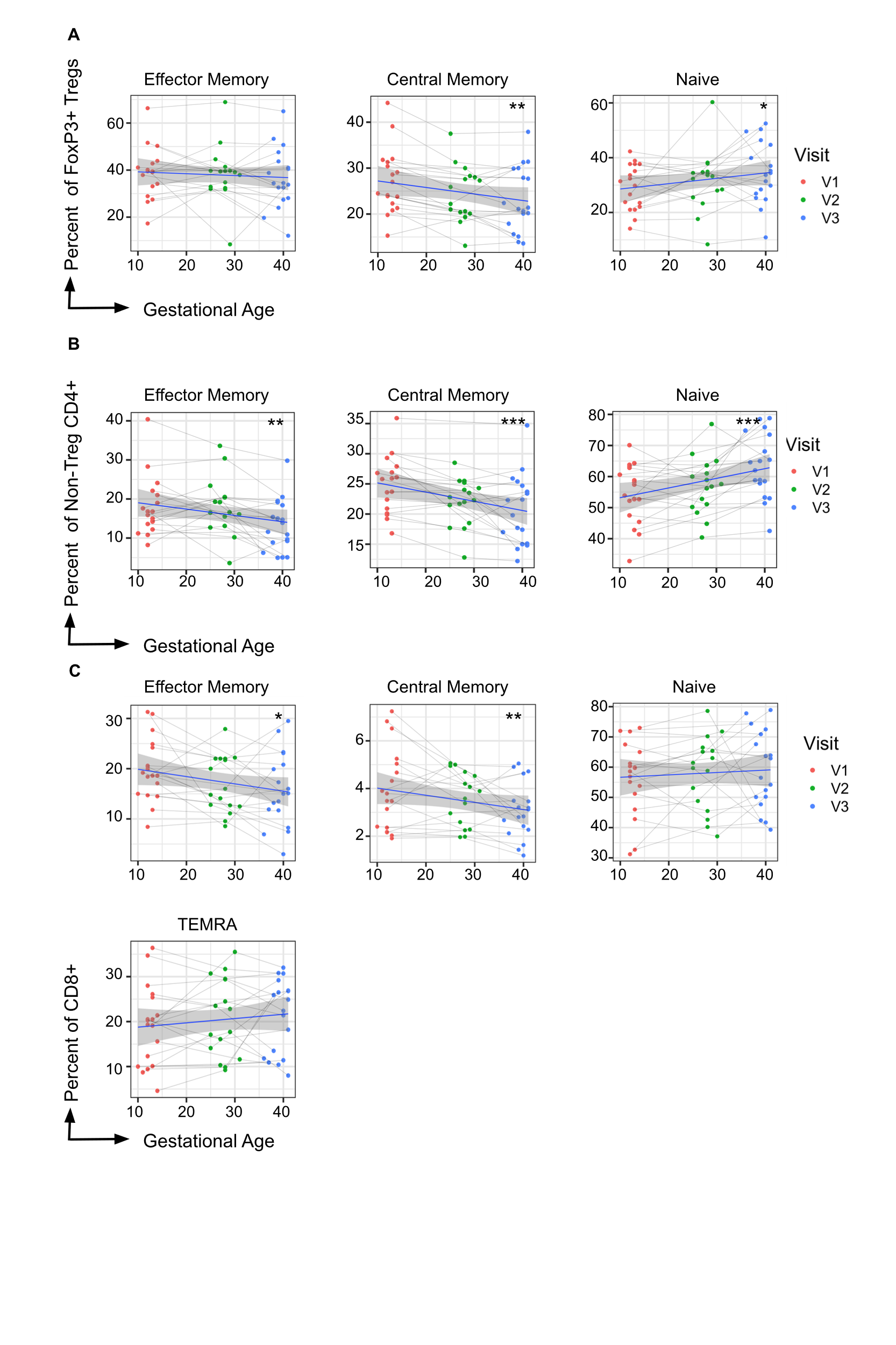


**Supplementary Figure 1.** **Peripheral FoxP3+ Tregs and Non-Treg CD4 T cells are dynamic over gestation**. Percent of effector memory, central memory, naïve, and TEMRA (CD8+ only) subpopulations of **(A)** FoxP3+ Tregs, **(B)** Non-Treg CD4+ T cells, and **(C)** CD8+ T cells is shown. Each data point represents an independent sample and a simple linear regression model (y~x) with a 95% CI is shown for visualization, with color indicating visit number. Changes across time were modeled using linear mixed-effects models, and p-values reflect the effect of gestational week on outcome shown on y-axes. *p ≤ 0.05, **p ≤ 0.01, **p ≤ 0.001.


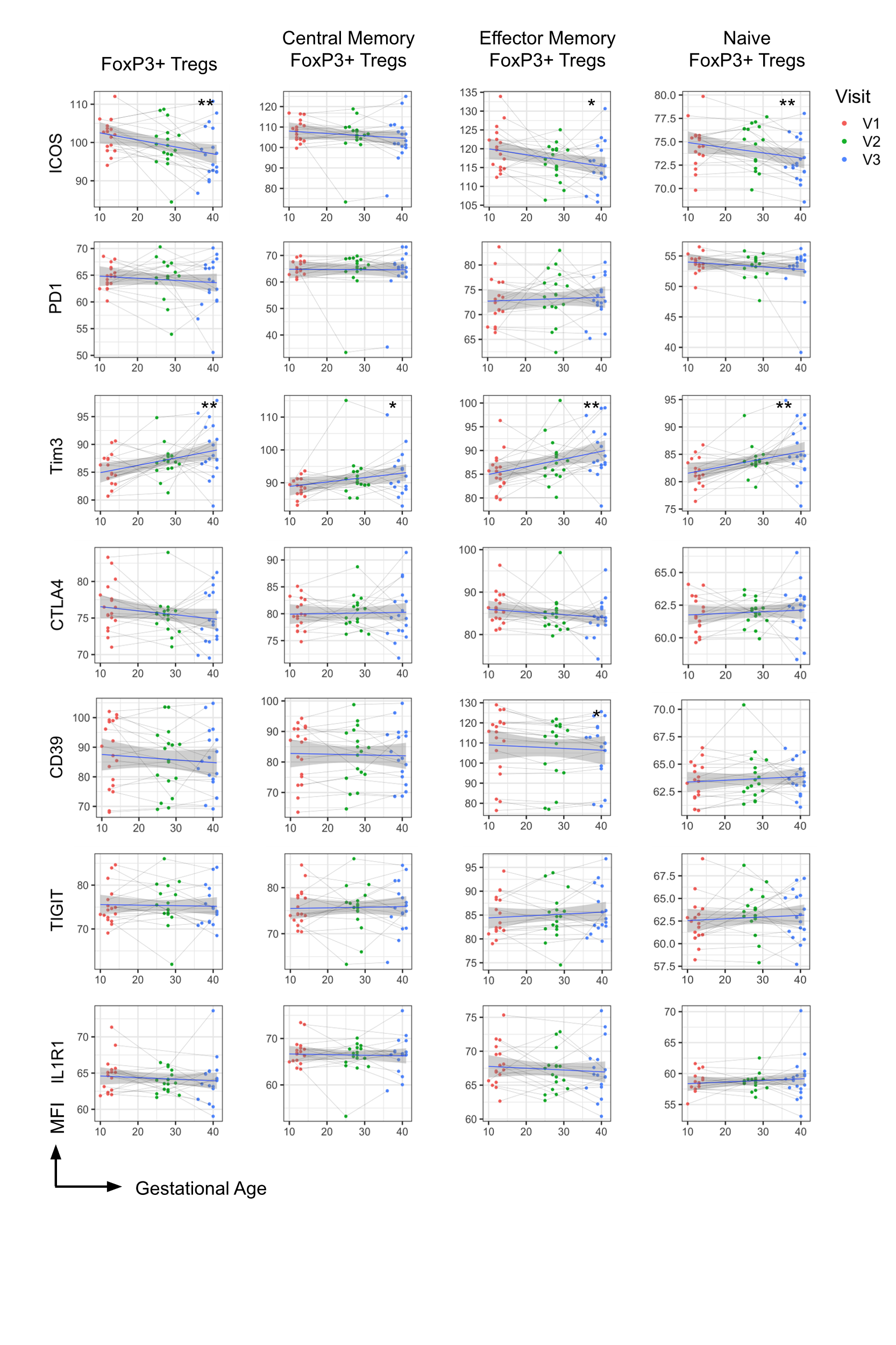


**Supplementary Figure 2. Most inhibitory markers do not change on peripheral FoxP3+ Tregs across gestation**. Mean fluorescent intensity (MFI) of IL1R1, TIGIT, CD39, CTLA4, Tim3, PD1 and ICOS on total FoxP3+ Tregs, central memory FoxP3+ Tregs, effector memory FoxP3+ Tregs, and naïve FoxP3+ Tregs are shown. Each data point represents an independent sample and simple linear regression model (y~x) with a 95% confidence interval is shown for visualization, with color indicating visit. Changes across time were modeled using linear mixed-effects models, and p-values reflect the effect of gestational week on outcome shown on y-axes. *p ≤ 0.05, **p ≤ 0.01, ***p ≤ 0.001.


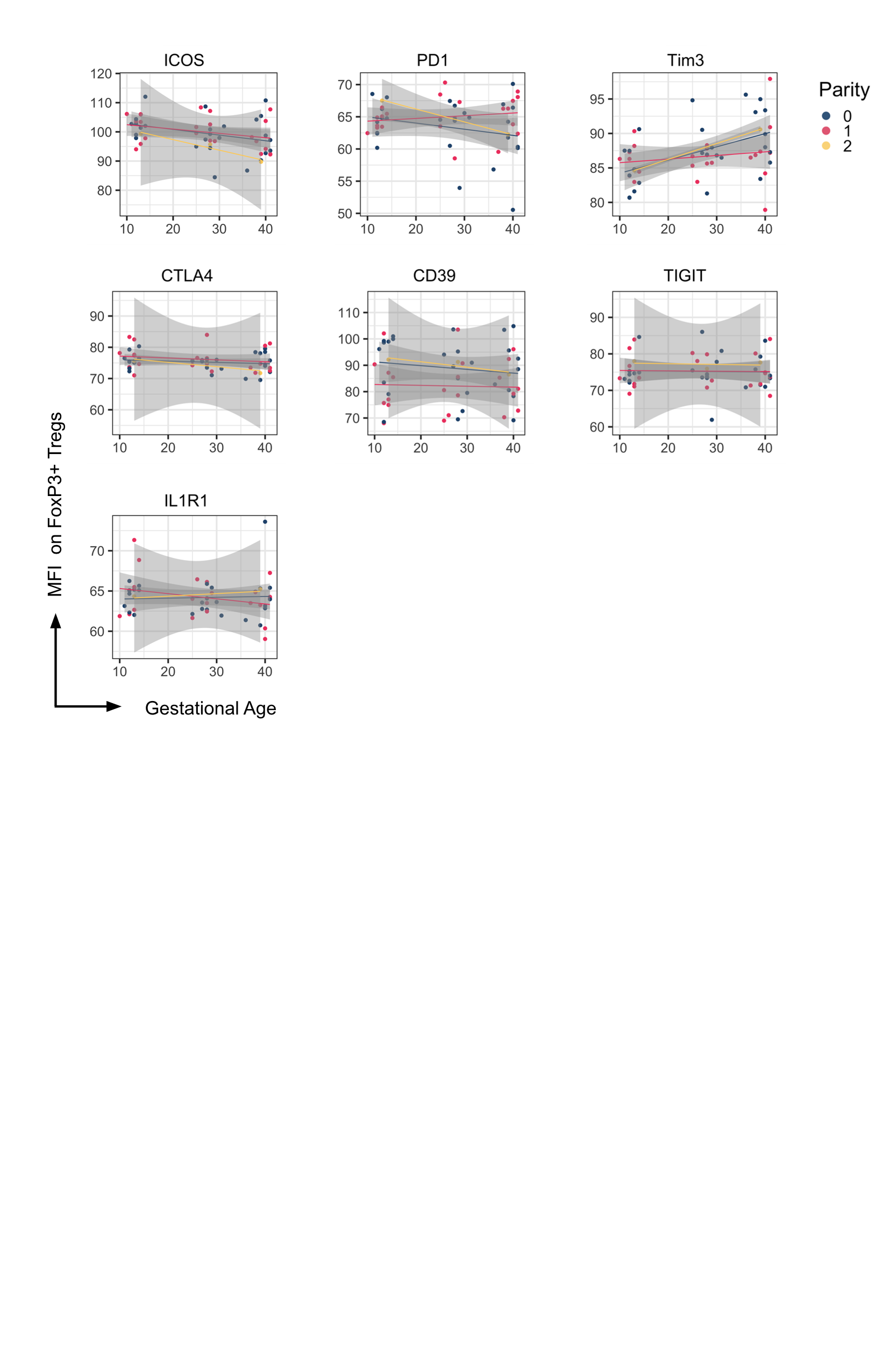


**Supplementary Figure 3. Parity does not influence expression of selected inhibitory markers on FoxP3+ Tregs**. Mean fluorescent intensity (MFI) of ICOS, PD1, Tim3, CTLA4, CD39, TIGIT and IL1R1 on FoxP3+ Tregs. Each data point represents an independent sample and simple linear regression model (y~x) with a 95% confidence interval is shown for visualization, with color indicating visit. Changes across time were modeled using linear mixed-effects models, and p-values reflect the effect of parity on outcome shown on y-axes.


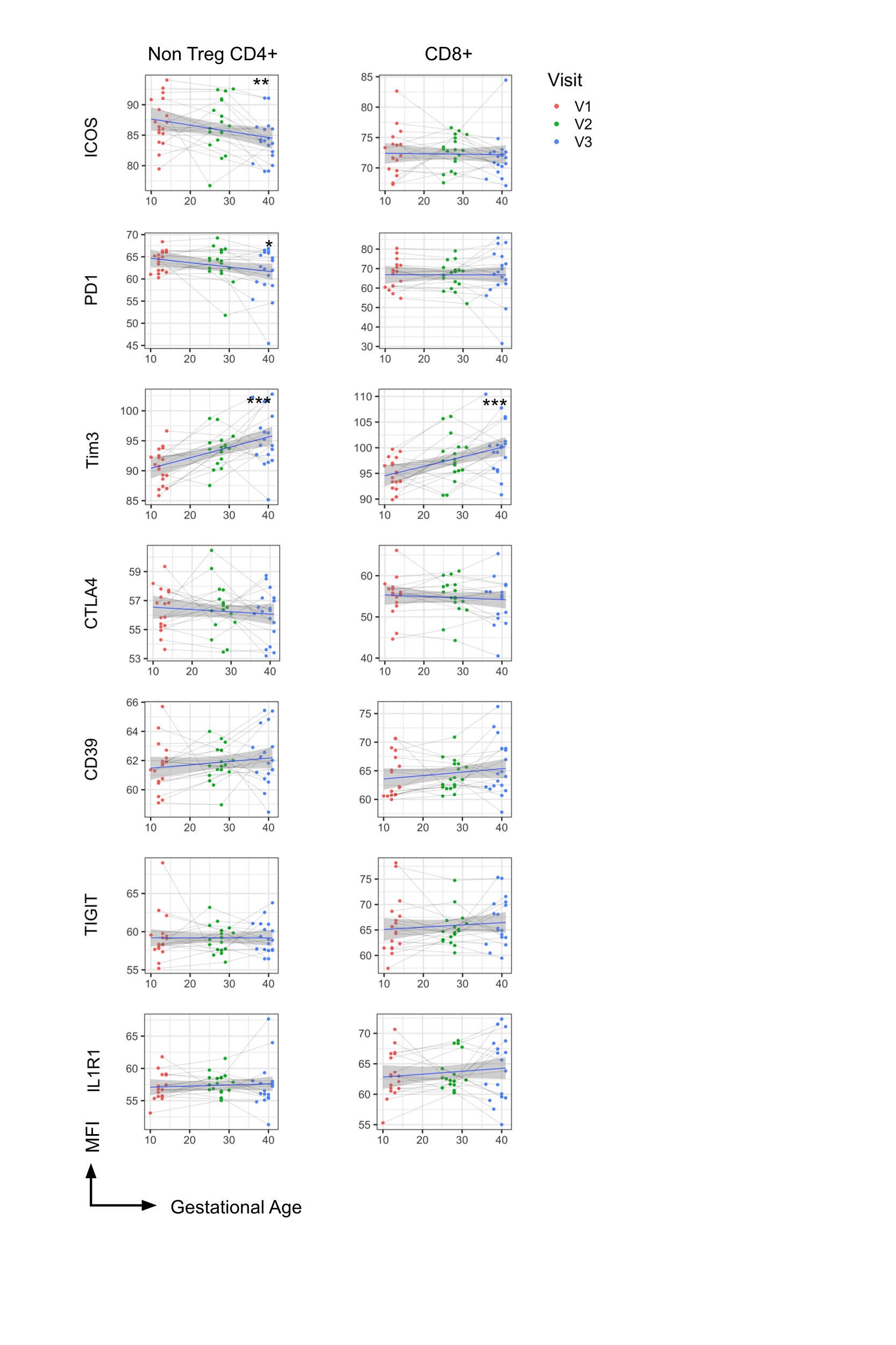


**Supplementary Figure 4. Expression of TIm3 increases on non Treg CD4+ and CD8+ T cells**. Mean fluorescent intensity (MFI) of ICOS, PD1, Tim3, CTLA4, CD39, TIGIT, and IL1R1 on non-Treg CD4+ T cells and CD8+ T cells. Each data point represents an independent sample and a simple linear regression model (y~x) with a 95% confidence interval is shown for visualization, with color indicating visit. Changes across time were modeled using linear mixed-effects models, and p-values reflect the effect of gestational week on outcome shown on y-axes. *p ≤ 0.05, **p ≤ 0.01, ***p ≤ 0.001


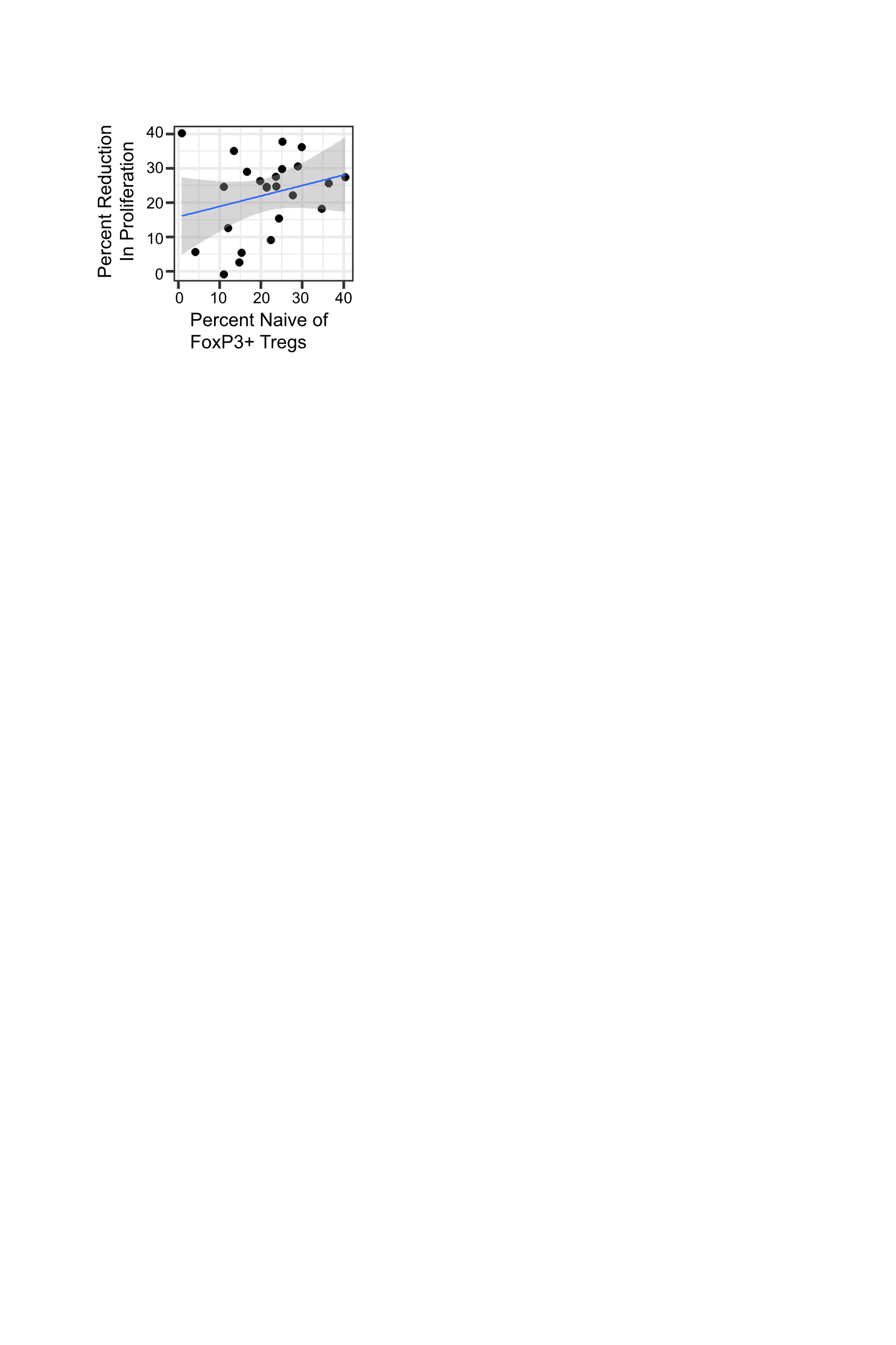
**Supplementary Figure 5. Treg suppressive correlates with frequency of Naïve Tregs in the Treg pool.** Frequency of naive Tregs (CD45RA+ CCR7+) of total FoxP3+ Tregs across gestation for selected participants (n=12) versus percent reduction in proliferation of Tresp with Tregs compared to Tresp alone for each participant and timepoint is shown. A simple linear regression model (y~x) with a 95% confidence interval is shown for visualization. *p ≤ 0.05, **p ≤ 0.01, ***p ≤ 0.001.

| **Laser** | **Fluorophore** | **Antigen** | **Annotation** |
| --- | --- | --- | --- |
| 355nm | BUV395 | CD8 | Lineage |
|  | L/D UV Blue | dead | Lineage |
|  | BUV496 | CD3 | Lineage |
|  | BUV563 | CD56 | Lineage |
|  | BUV661 | CCR7 | Antigen Experience |
|  | BUV737 | ICOS | Inhibitory/Checkpoint |
| 405nm | BV421 | CD25 | Lineage |
|  | BV510 | GrzmB | Cytotoxicity |
|  | BV570 | CD45RA | Antigen Experience |
|  | BV605 | CD39 | Inhibitory/Checkpoint |
|  | BV650 | CD69 | Activation |
|  | BV711 | CD14 | Lineage |
|  | BV750 | CD103 | Tissue Residence |
|  | BV785 | KI67 | Proliferation |
| 488nm | BB515 | Tim3 | Inhibitory/Checkpoint |
|  | BB630 | CTLA4 | Inhibitory/Checkpoint |
|  | BB660 | CD127 | Lineage |
|  | BB700 | PD1 | Inhibitory/Checkpoint |
|  | BB790 | TIGIT | Inhibitory/Checkpoint |
| 532nm | PE | IL1R1 | Inhibitory/Checkpoint |
|  | PEeFlour610 | EOMES | Cytotoxicity |
|  | PE-Cy5 | CD137 | Activation |
|  | PE-Cy5.5 | Foxp3 | Lineage |
|  | PE-Cy7 | Tbet | Cytotoxicity |
| 628nm | APC | TOX | Exhaustion |
|  | AF700 | TCF1 | Stemness |
|  | APC-H7 | CD4 | Lineage |

**Supplementary Table 1. 27 color flow phenotyping panel**

| **Type (number)** | **Condition - Count** |
| --- | --- |
| Prior pregnancy conditions (n=14) | None - 5 |
|  | Spontaneous Abortion - 7 |
|  | Preterm Delivery - 1 |
|  | Gestational Diabetes - 1 |
| Preexisting health conditions (n=18) | None - 16 |
|  | Hashimoto's disease - 1 |
|  | Chronic Hypertension - 1 |
| Current Pregnancy Conditions (n=18) | None - 16 |
|  | Gestational Diabetes - 2 |

**Supplementary Table 2**. **Participant characteristics**
